# Complete online database of maximal subgroups of subperiodic groups at the Bilbao Crystallographic Server

**DOI:** 10.1107/S1600576725001499

**Published:** 2025-03-13

**Authors:** Gemma de la Flor, Hans Wondratschek, Mois I. Aroyo

**Affiliations:** a Karlsruhe Institute of Technology, Institute of Applied Geosciences, Karlsruhe, Germany; b Laboratorium für Applikationen der Synchrotronstrahlung (LAS), Universität Karlsruhe, Germany; c Departamento de Física Universidad del País Vasco UPV/EHU Spain; Shiv Nadar Institution of Eminence, India

**Keywords:** subperiodic groups, maximal subgroups, Bilbao Crystallographic Server, series of maximal subgroups

## Abstract

The section of the Bilbao Crystallographic Server (https://www.cryst.ehu.es) dedicated to subperiodic groups includes the program *MAXSUB*, which gives online access to the complete database of maximal subgroups of subperiodic groups. All maximal non-isotypic subgroups as well as all maximal isotypic subgroups of indices up to 9 are listed individually, together with the series of maximal isotypic subgroups of subperiodic groups. These data were compared with those of Litvin [(2013), *Magnetic group tables, 1-, 2- and 3-dimensional subperiodic groups and magnetic space groups*], which revealed several differences, discussed here in detail.

## Abstract 2

The complete maximal subgroups of subperiodic groups database of the Bilbao Crystallographic Server is presented. The program *MAXSUB* gives online access to all maximal non-isotypic as well as all maximal isotypic subgroups of indices up to 9, and to the series of maximal isotypic subgroups of subperiodic groups.

## 1. Introduction

Crystallographic information about space groups is published in *International tables for crystallography*, Vol. A, *Space-group symmetry* (Aroyo, 2016 ▸; henceforth abbreviated as *IT*A). The complete listing of the maximal subgroups of all 230 space groups, however, is available in *International tables for crystallography*, Vol. A1, *Symmetry relations between space groups* (Wondratschek & Müller, 2010 ▸; henceforth abbreviated as *IT*A1). Aside from the subgroups of space groups with three-dimensional lattices which are again space groups, there also exist subgroups called subperiodic groups with translation lattices of dimensions one or two. These are the groups required to describe polymers, nanotubes, nanowires and layered materials (Müller, 2017 ▸; Gorelik *et al.*, 2021 ▸; de la Flor & Milošević, 2024 ▸).

The interest in materials with subperiodic symmetry is constantly growing due to their outstanding properties and possible technological applications (Xu *et al.*, 2013 ▸). There are three types of subperiodic groups: *frieze groups* (two-dimensional groups with one-dimensional translation lattices), *rod groups* (three-dimensional groups with one-dimensional translation lattices) and *layer groups* (three-dimensional groups with two-dimensional translation lattices). Frieze groups do not correspond to any physical atomic structure, as real objects cannot be strictly confined to a two-dimensional space. While they are useful for describing physical properties and geometric patterns, they have no direct application to real structures. The crystallographic data for subperiodic groups are compiled in *International tables for crystallography*, Vol. E, *Subperiodic groups* (Kopský & Litvin, 2010 ▸; henceforth referred to as *IT*E). Since there is not a volume in *International tables for crystallography* for subperiodic groups similar to *IT*A1, the maximal subgroups of subperiodic groups are listed in *IT*E. This listing follows the format of *IT*A (Hahn, 2002 ▸) but lacks additional information, such as a complete list of maximal subgroups. It also omits the series of maximal isotypic subgroups of subperiodic groups, where isotypic refers to subgroups belonging to the same *subperiodic group type*. (One often refers to the layer, rod and frieze groups without distinguishing between the terms layer group type, rod group type and frieze group type. In many cases, this distinction is not necessary, and in order to avoid unnecessarily lengthy terminology, the same approach is taken in this article.) Additionally, the minimal supergroups are not included in *IT*E. To the best of our knowledge, the only complete compilation of maximal subgroups of subperiodic groups, but only of indices up to 4, can be found in *Magnetic group tables, 1-, 2- and 3-dimensional subperiodic groups and magnetic space groups* (Litvin, 2013 ▸; henceforth referred to as Litvin’s book), an electronic book of about 12000 pages. However, the series of maximal isotypic subgroups of subperiodic groups are also not available.

The complete data about the maximal subgroups of subperiodic groups are now available online in the databases of the Bilbao Crystallographic Server (https://www.cryst.ehu.es) (Aroyo *et al.*, 2011 ▸; Tasci *et al.*, 2012 ▸; hereafter referred to as BCS), in the section *Subperiodic groups: layer, rod and frieze groups*. In contrast to *IT*E, the BCS database of maximal subgroups of subperiodic groups provides the complete listing (not just by type but individually) of all maximal non-isotypic and all maximal isotypic subgroups of subperiodic groups of indices up to 9. The list of maximal subgroups is retrieved by the program *MAXSUB*, which also gives access to the series of maximal isotypic subgroups of subperiodic groups.

The aim of this contribution is to present the complete database of maximal subgroups and series of maximal isotypic subgroups of subperiodic groups available in the BCS. The procedure applied to derive the maximal subgroups of subperiodic groups is described in Section 3. The data from Litvin’s book were reviewed and compared with those from the BCS, and their differences are listed in Section 5 in detail.

## 2. Subperiodic groups

Subperiodic groups are two- and three-dimensional groups with one- and two-dimensional translations. The 80 layer groups together with the 75 rod groups and the seven frieze groups constitute the subperiodic groups. The section *Subperiodic groups: layer, rod and frieze groups* of the BCS hosts the subperiodic groups crystallographic databases. The structure of these databases is similar to that of the space groups – they include information on generators, general positions, Wyckoff positions and maximal subgroups for subperiodic groups. Apart from the data shown in *IT*E, the server offers additional information and computer tools that allow the generation of data not available in *IT*E. The BCS also hosts the Brillouin-zone database for layer groups (de la Flor *et al.*, 2021 ▸) and more complex programs to calculate, for example, the site-symmetry induced representations of layer groups (de la Flor *et al.*, 2019 ▸). Note that in the programs of the BCS the Hermann–Mauguin symbols for frieze and rod groups do not use the calligraphy font used in *IT*E to depict the Bravais-lattice type, *i.e.* the frieze group 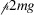 (No. 7) and the rod group 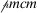 (No. 22) are represented as *p2mg* and 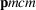 in the BCS, respectively.

The programs and databases of the BCS related to subperiodic groups use the standard or default settings of the subperiodic groups. These are the specific settings of subperiodic groups that coincide with the conventional subperiodic group descriptions found in *IT*E. For layer groups with more than one description in *IT*E, the following settings are chosen as standard: (i) *cell-choice 1* description for the two monoclinic/oblique layer groups *p*11*a* (No. 5) and 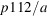 (No. 7) given with respect to three cell choices in *IT*E, and (ii) *origin choice 2* descriptions (*i.e.* when the origin is at a centre of inversion) for the three layer groups 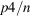 (No. 52), 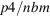 (No. 62) and 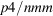 (No. 64) listed with respect to two origins in *IT*E. For rod groups, the first setting is chosen as standard for the trigonal and hexagonal groups with two descriptions (*cf.* Table 1.2.6.3 of *IT*E).

Following the conventions of *IT*E, the *ab* plane is the plane of periodicity for layer groups; this means that the translation vectors are of the form 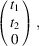
where 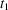 and 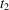 are integer numbers.

For rod groups, the *c* axis is the line of periodicity and the translation vectors are of the form 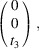
where 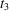 is an integer number.

In the case of frieze groups, the periodicity is along the *a* axis; therefore, the translation vectors are of the form 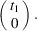


As in space groups, for subperiodic groups a group–subgroup pair 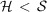 is also characterized by the group 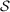, subgroup 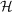, index 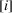 and transformation matrix–column pair (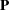, 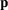) relating the basis of 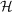 and 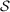. The matrix–column pair (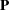, 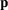) describes a coordinate transformation and consists of two parts:

(i) A linear part 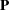, denoted by a (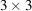) matrix for rod and layer groups and by a (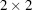) matrix for frieze groups, describing the change of direction and/or length of the basis vectors: 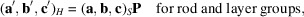

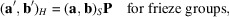
where 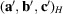 and 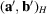 represent the bases of the subgroup 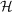 and 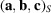 and 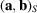 the bases of the subperiodic group 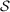.

(ii) An origin shift 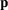 denoted by a (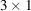) column vector 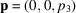 for rod groups and 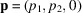 for layer groups; and by a (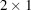) column vector 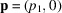 for frieze groups. The coefficients of 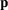 describe the position of the origin 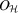 of 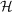 referred to the coordinate system of 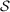.

The data of the matrix–column pair (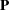, 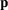) are often written in the following concise form for rod and layer groups:
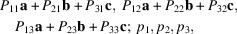
where 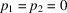 for rod groups and 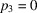 for layer groups. For frieze groups, the form is
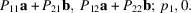


## 3. Derivation of the maximal subgroups of subperiodic groups based on the group–subgroup relations between subperiodic and space groups

A group–subgroup relationship exists between subperiodic groups 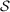 and space groups 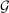, *i.e.*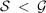. For each subperiodic group, there is a two- or three-dimensional space group 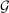 with the same symmetry diagram and general-position diagram. These relationships have been considered in detail in the literature [see *e.g.* Wood (1964 ▸), *IT*E and references therein]. The type of space group of which a given subperiodic group is a subgroup is not defined uniquely. The ‘simplest’ space group 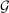 to which 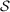 is related can be expressed as a semi-direct product of 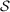 with a one- or two-dimensional translation group 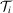 of additional translations 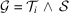, where 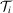 is a normal subgroup of 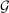 (Evarestov & Smirnov, 1993 ▸; Smirnov & Tronc, 2006 ▸). Thus, subperiodic groups 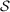 are isomorphic to factor groups 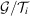 (Litvin & Kopský, 1987 ▸, 2000 ▸). In the case of layer groups 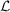 (defined as a three-dimensional crystallo­graphic group with periodicity restricted to a two-dimensional subspace), the three-dimensional space group 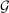 to which a layer group 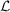 is related can be expressed as a semi-direct product of 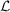 with the one-dimensional translation group 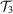 of additional translations 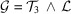. As a result of this, the layer group 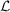 is isomorphic with the factor group 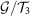. For rod groups 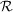 (defined as a three-dimensional crystallographic group with periodicity restricted to a one-dimensional sub­space), the three-dimensional group 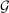 to which a rod group 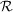 is related can be represented as a semi-direct product of 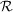 and the two-dimensional translation group 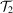 of additional translations 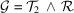. This means that the rod group 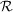 is isomorphic with the factor group 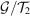. Finally, for frieze groups 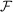 (defined as a two-dimensional crystallographic group with periodicity restricted to a two-dimensional subspace), the two-dimensional space group 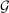 (plane group) to which a frieze group 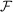 is related can be expressed as a semi-direct product of 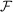 with the one-dimensional translation group 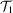 of additional translations 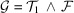. Therefore, the frieze group 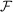 is isomorphic with the factor group 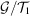.

The isomorphism between the subperiodic group 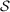 and the factor group 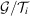 results in a close relationship between the Wyckoff positions, maximal subgroups, minimal supergroups and irreducible representations of 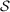 and 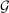. For example, one can show that the set of Wyckoff positions of a subperiodic group is contained in the set of Wyckoff positions of the related space (or plane) group (*cf.* Evarestov & Smirnov, 1993 ▸). The restrictions imposed by the loss of periodicity result in the restrictions of the special-position coordinates of subperiodic groups.

The maximal subgroups of subperiodic groups 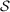 can be derived from the maximal subgroups of the two- or three-dimensional space groups, since the set of maximal subgroups of a subperiodic group is contained in the set of maximal subgroups of the related space group. The maximal subgroups database for subperiodic groups was constructed from the maximal subgroups database of two- and three-dimensional space groups provided by the BCS (Aroyo *et al.*, 2006 ▸). These subgroups were classified into two types: *translationengleiche* and *klassengleiche* subgroups (for further details, see Appendix *A*[App appa]). Additionally, the classification of maximal subgroups of subperiodic groups into conjugacy classes can be derived from the corresponding classification for space groups. Consider the subgroups 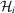 and 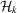, which are subgroups of the space group 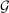 and the subperiodic group 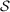 (where 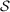 is isomorphic to the factor group 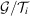). These two subgroups, 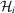 and 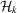, are said to be conjugate if there exists an element *g* of the space group 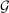 such that 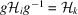. Furthermore, if *g* is an element of the subperiodic group 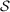, then 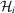 and 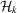 remain conjugate subgroups within 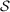 as well. As an example, let us determine the maximal subgroups of indices up to 4 for the layer group *p*4 (No. 49) and the rod group 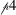 (No. 23), isomorphic to factor groups 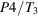 and 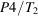, respectively. Table 1 ▸ shows the maximal subgroups of indices up to 4 for the space group *P*4 (No. 75). The loss of periodicity along the *z* direction restricts the maximal subgroups of layer groups: only the maximal subgroups of 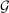 without loss of translations along the *c* axis are maximal subgroups of the layer groups. In this case, there are three maximal subgroups for the layer group *p*4: one *translationengleiche* subgroup *p*112 (No. 3) of index 2 and transformation matrix (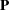, 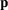) = 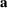, 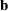, 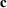, and two *klassengleiche* subgroups *p*4 (No. 49) of index 2 and transformation matrices 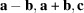 and 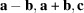; 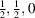.

The loss of periodicity along the *x* and *y* directions restricts the maximal subgroups of rod groups: only the maximal subgroups of 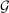 without loss of translations along the *a* and *b* axes are maximal subgroups of the rod groups. Therefore, for the rod group 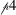 (No. 23) there are four maximal subgroups: (i) the *translationengleiche* subgroup 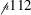 (No. 8) of index 2 and (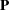, 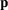) = 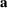, 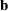, 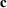; (ii) the *klassengleiche* subgroup 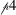 (No. 23) of index 2 and (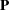, 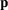) = 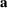, 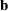, 2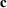; (iii) the *klassengleiche* sub­group 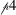 (No. 23) of index 3 and (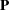, 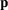) = 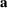, 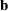, 3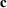; and (iv) the *klassengleiche* subgroup 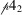 (No. 25) of index 2 and (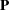, 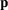) = 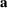, 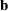, 2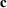.

The rest of the maximal subgroups of subperiodic groups of indices up to 9 were calculated from the series of maximal isotypic subgroups of subperiodic groups. These series can also be directly derived from the series of maximal isomorphic subgroups of space groups. For example, for the space-group type *P*4 there are three series of maximal isomorphic subgroups (see Table 2 ▸). Two of these series, Series #2 and #3, are also the series of maximal isotypic subgroups for the layer group *p*4 since the loss of translations occurs in the *ab* plane. For the rod group 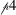, however, only one series of maximal isotypic subgroups exists with loss of translations along the *c* axis; this corresponds to the Series #1 in Table 2 ▸.

## 4. The program *MAXSUB*

The database of the maximal subgroups of subperiodic groups of the BCS is accessible from the *MAXSUB* program (https://www.cryst.ehu.es/subperiodic/get_sub_maxsub.html) in the section *Subperiodic groups: layer, rod and frieze groups*. This provides the complete listing of (i) all maximal non-isotypic subgroups for each subperiodic group, and (ii) all maximal isotypic subgroups of indices up to 9. In addition to this, there is also an option in the program to retrieve the series of maximal isotypic subgroups.

**Figure 1:**
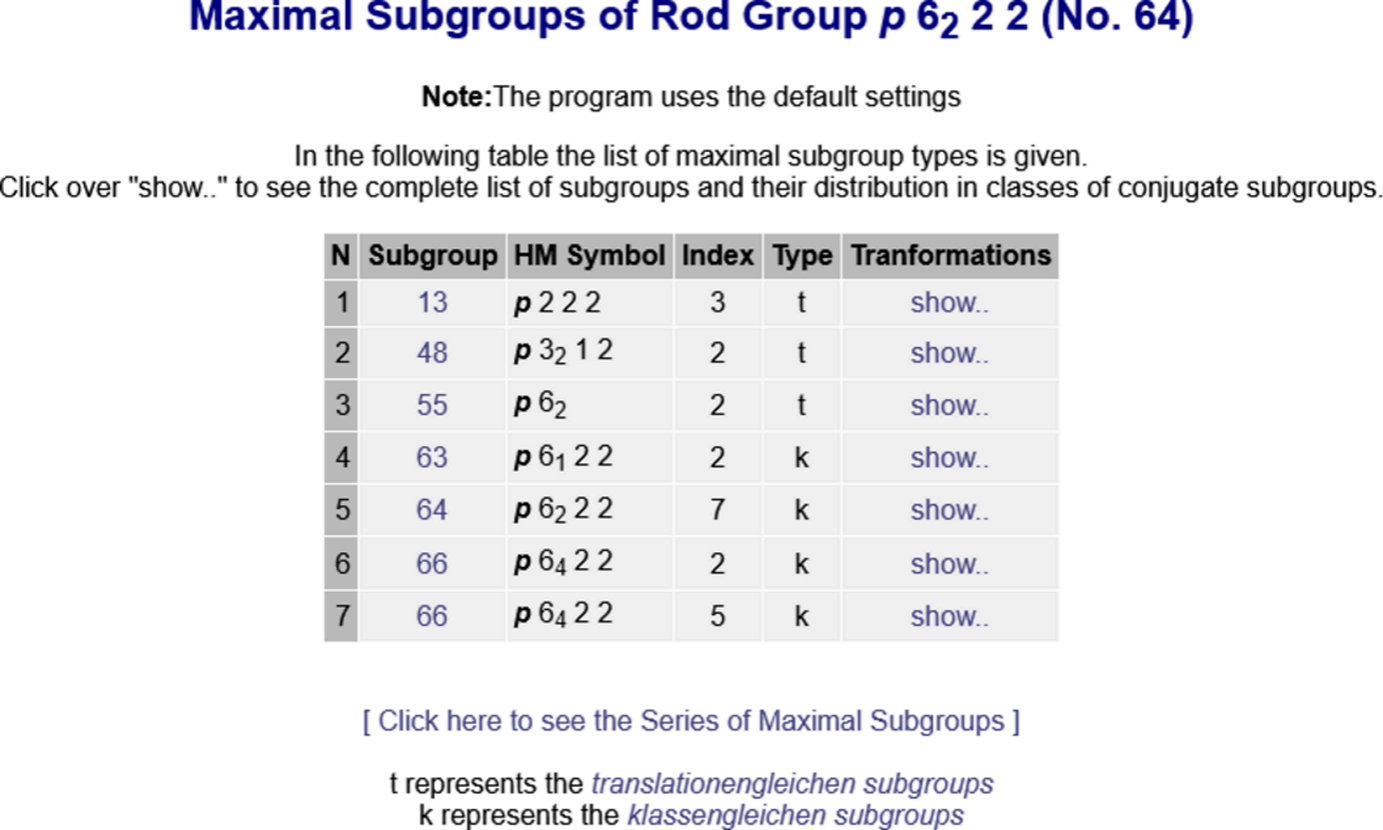
List of maximal subgroups of the rod group 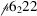 (No. 64) as displayed by the program *MAXSUB*. Subgroups marked as *t* and *k* correspond to *translationengleiche* and *klassengleiche* subgroups, respectively. Clicking on ‘*show..*’ reveals the complete list of subgroups and their distribution in classes of conjugate subgroups (see Fig. 2 ▸). The link ‘*Click here to see the Series of Maximal Subgroups*’ gives direct access to the maximal isotypic subgroups of the rod group 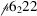 (see Fig. 3 ▸). Note that the Hermann–Mauguin symbols for rod groups in the BCS do not use the calligraphy font used in *IT*E to depict the Bravais-lattice type.

**Figure 2:**
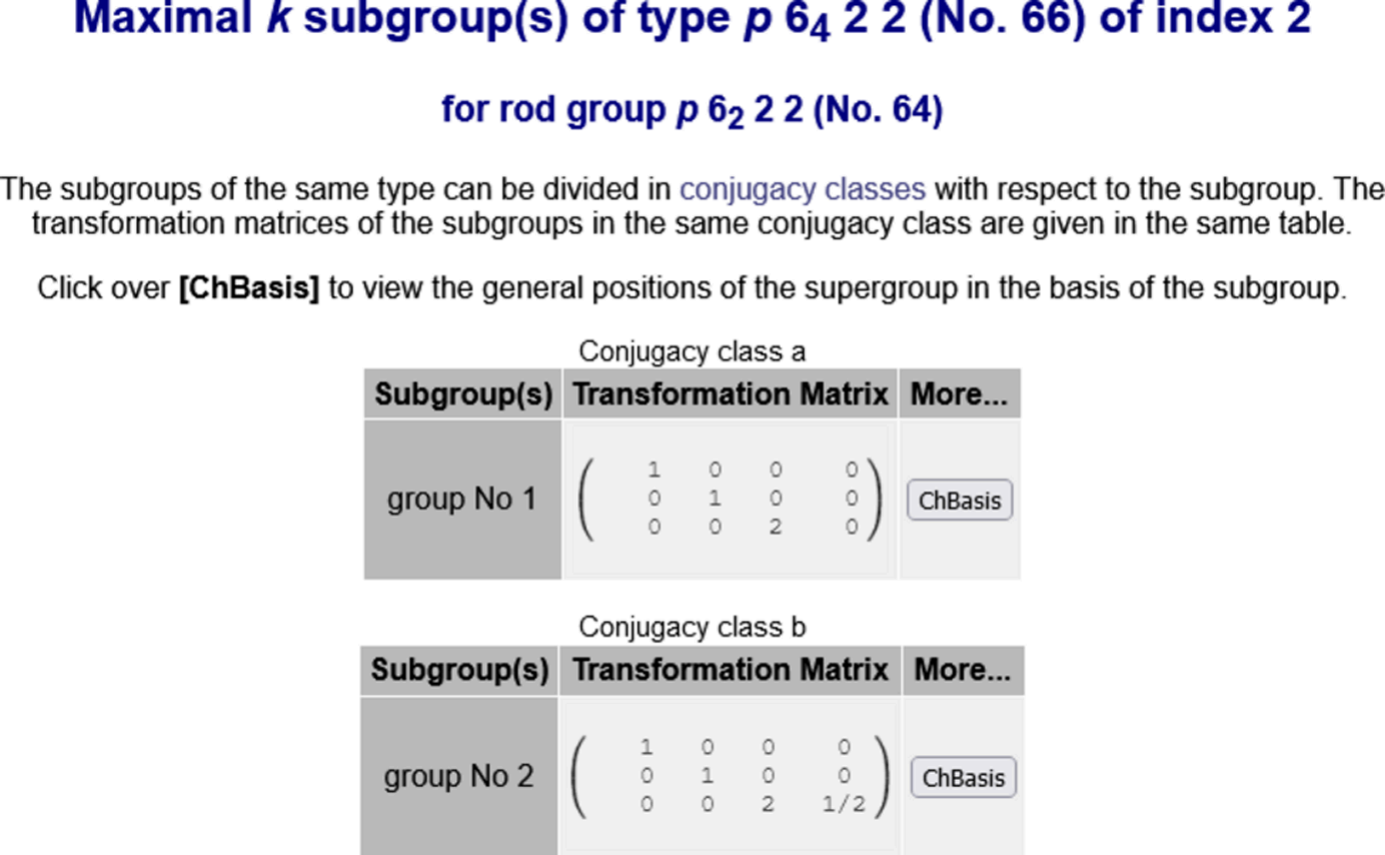
The maximal *klassengleiche* subgroups 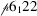 (No. 63) of index 2 for the rod group 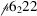 (No. 64) obtained by clicking on ‘*show..*’ in Fig. 1 ▸. There are two subgroups for 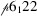 distributed in two conjugacy classes. Note that the Hermann–Mauguin symbols for rod groups in the BCS do not use the calligraphy font used in *IT*E to depict the Bravais-lattice type.

The subperiodic-group type (frieze, rod or layer) and the corresponding *IT*E number of the group are required as input to the program *MAXSUB*. If the *IT*E number is unknown, this can be selected from a table with the Hermann–Mauguin symbols of the selected subperiodic-group type. The program first returns a table with the maximal subgroup 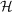 of the selected subperiodic group 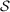 (see Fig. 1 ▸). Each subgroup 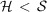 is specified by its *IT*E number, Hermann–Mauguin symbol, index and subgroup type (*t* for *translationengleiche* or *k* for *klassengleiche* subgroup, see Appendix *A*[App appa] and Section 2.2.4 of *IT*A1). The complete list of subgroups and their distribution in classes of conjugate subgroups is obtained by clicking on the link ‘*show..*’. For example, the rod group 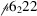 (No. 64) has two maximal *klassengleiche* subgroups 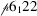 (No. 63) of index 2 distributed in two conjugacy classes of conjugate subgroups (see Fig. 2 ▸). The transformation matrix–column pairs (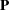, 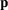) that relate the standard basis of 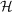 and 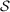 are also provided by the program.

For certain applications, it is necessary to represent the subgroups 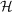 as subsets of the elements of 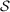. This is achieved by the option ‘*ChBasis*’ (see Fig. 2 ▸), which transforms the general position of 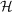 to the coordinate system of 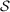.

**Figure 3:**
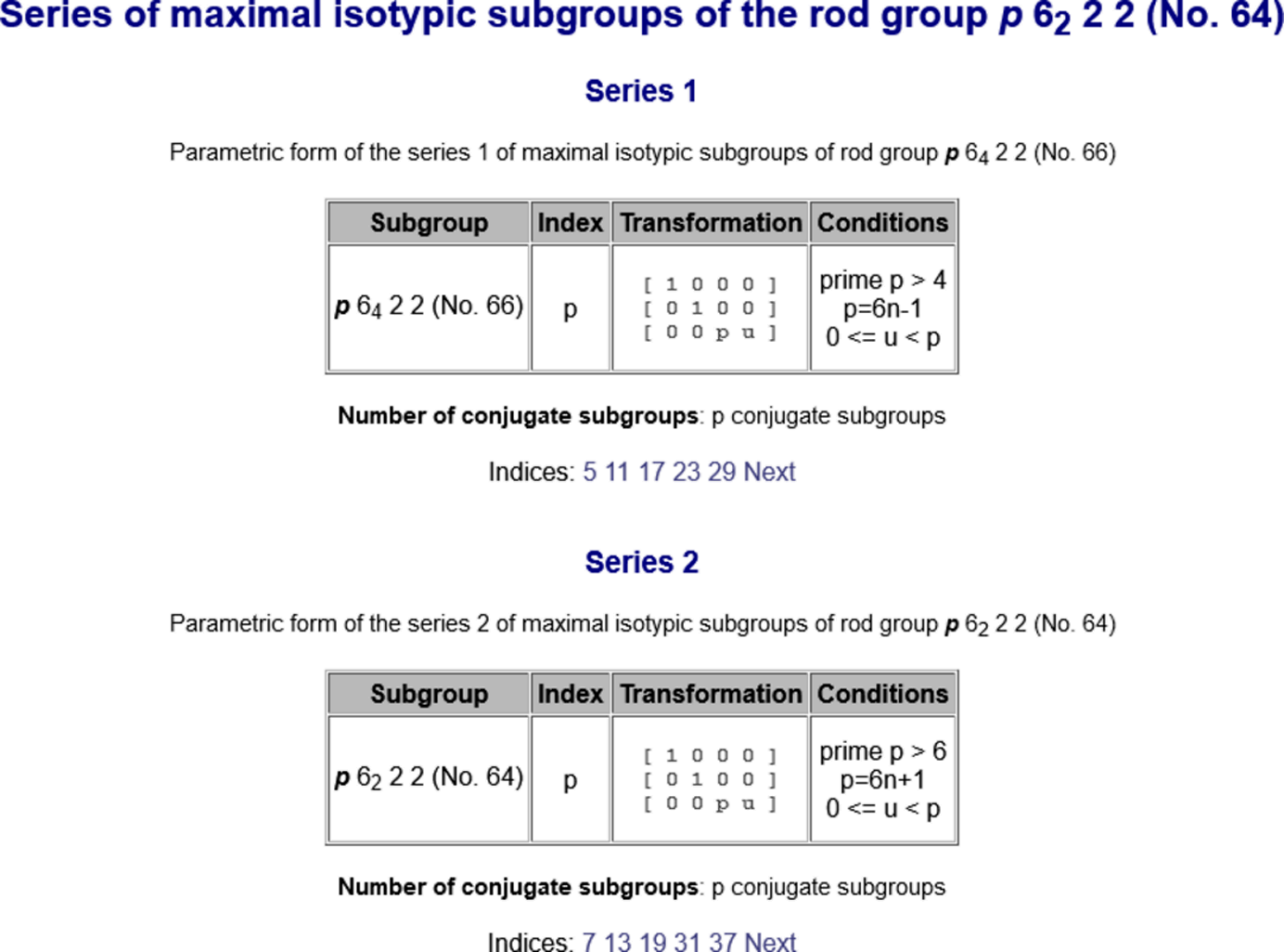
Output of the program *MAXSUB* showing the two series of maximal isotypic subgroups for the rod group 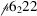 (No. 64). Since the rod group 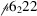 belongs to one of the eight pairs of enantiomorphic rod groups, the subgroup of Series 1 corresponds to its enantiomorphic pair 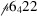 (No. 66). When the user clicks on the indices below the tables, the program is able to generate the maximal isotypic subgroups for the chosen index (see Fig. 4 ▸). Note that the Hermann–Mauguin symbols for rod groups in the BCS do not use the calligraphy font used in *IT*E to depict the Bravais-lattice type.

Maximal subgroups of index higher than 4 have indices *p* for frieze and rod groups, and *p* and 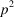 for layer groups, where *p* is a prime. These are isotypic subgroups and are infinite in number. In most of the series, the Hermann–Mauguin symbol for each isotypic subgroup is the same. However, if the subperiodic group belongs to a pair of enantiomorphic groups, the Hermann–Mauguin symbol of the isotypic subgroup is either that of the group or that of its enantiomorphic pair (see Fig. 3 ▸). Note that among the subperiodic groups there are only eight pairs of enantiomorphic rod groups: 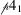 (No. 24), 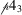 (No. 26); 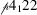 (No. 31), 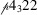 (No. 33); 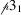 (No. 43), 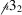 (No. 44); 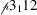 (No. 47), 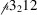 (No. 48); 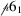 (No. 54), 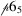 (No. 58); 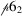 (No. 55), 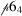 (No. 57); 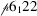 (No. 63), 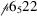 (No. 67); and 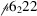 (No. 64), 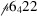 (No. 66).

**Figure 4:**
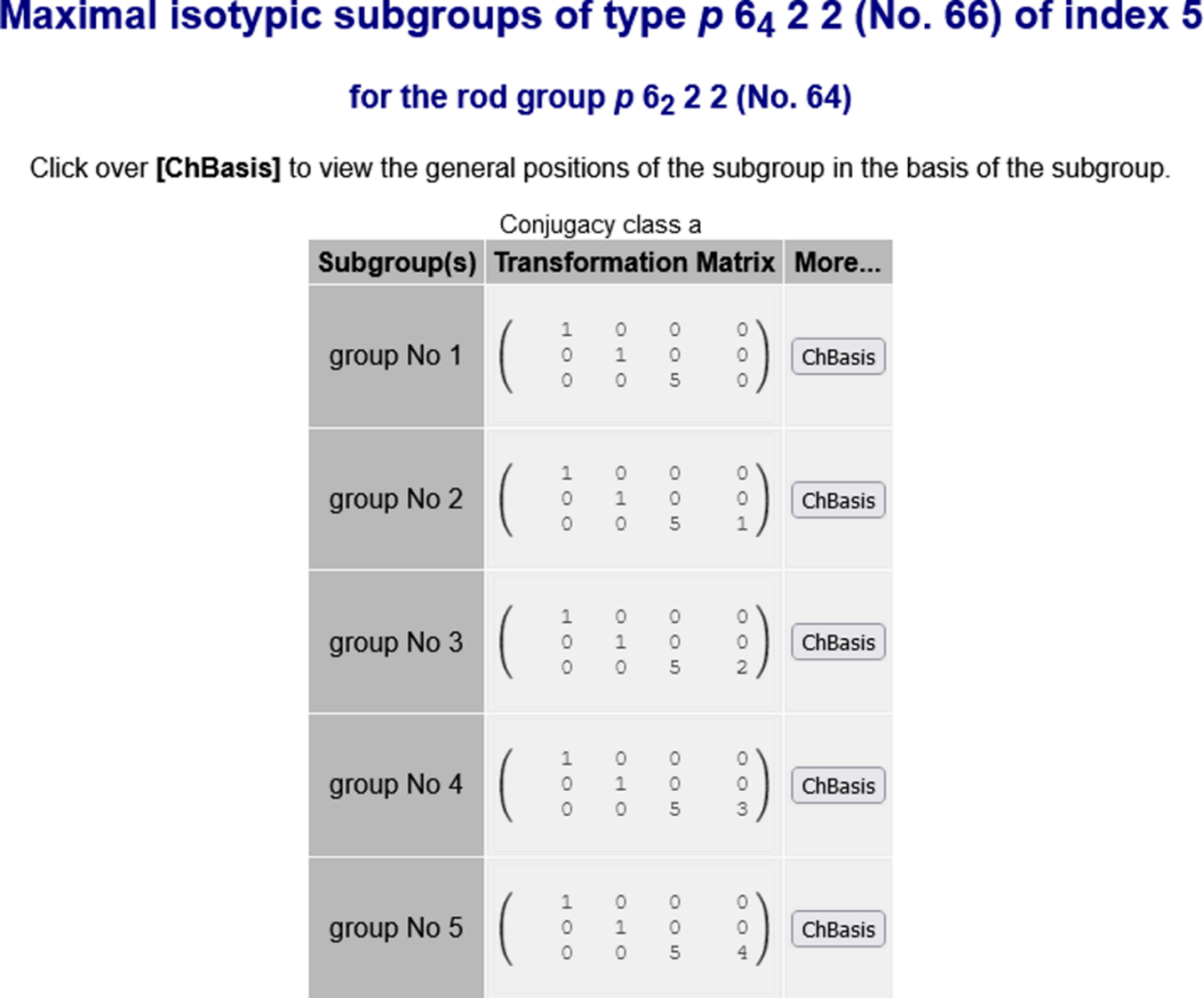
Complete list of the series of maximal isotypic subgroups 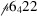 (No. 66) of index 5 for the rod group 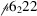 (No. 64) generated by the auxiliary tool of the program *MAXSUB* (see Fig. 3 ▸). Note that the Hermann–Mauguin symbols for rod groups in the BCS do not use the calligraphy font used in *IT*E to depict the Bravais-lattice type.

There is a link in the program *MAXSUB* (see Fig. 1 ▸) that gives direct access to the series of maximal isotypic subgroups of subperiodic groups. Apart from the parametric descriptions of the series, the program provides the individual listing of all maximal isotypic subgroups. The series of maximal isotypic subgroups are shown in blocks grouped by the index and the transformation matrix–column pair (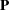, 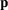) (see Fig. 3 ▸). For each series, the Hermann–Mauguin symbol of the subgroup, the restrictions on the parameters describing the series, and the transformation matrix (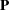, 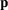) relating the group 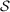 and the subgroup 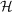 are listed. As an example, Fig. 3 ▸ shows the output of maximal isotypic subgroups for the rod-group type 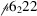 (No. 64), which is subdivided into two series. There is a special tool that permits the online generation of maximal isotypic subgroups of any allowed index. Fig. 4 ▸ shows the series of maximal isotypic subgroups 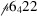 (No. 66) of index 5 for the rod-group type 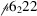 (No. 64) generated by this auxiliary tool.

## 5. Differences between Litvin’s book and the BCS maximal subgroups of subperiodic groups database

Litvin’s book gives the complete listing of the maximal subgroups 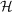 of subperiodic groups 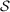 of indices up to 4. For each maximal subgroup 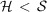, the Hermann–Mauguin symbol, the index, the transformation relating the setting of the subperiodic group 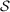 to the setting of the group 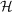 and the coset representatives (in Seitz notation) of the coset decomposition of 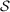 relative to 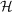 are specified. Note that in Litvin’s book the standard International Union of Crystallography Seitz notation is not followed, *e.g.* a twofold rotation around the *c* axis is denoted by 2_*z*_ instead of 2_001_ [for details *cf.* Litvin & Kopský (2014 ▸)].

The maximal subgroups of subperiodic groups of indices up to 4 of the BCS were compared with a subset of the tables in Litvin’s book. As a result of this comparison, some differences were detected for the maximal subgroups of rod and layer groups; no differences were found for frieze groups. Several errors were identified in Litvin’s book (for more details, see Tables 3 to 6). This list of discrepancies was reviewed with D. Litvin, who has acknowledged them (Litvin, personal communication).

### Transformation matrix (**P**, **p**) relating the basis of 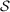 and 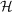


5.1.

The main difference between Litvin’s book and the BCS is in the transformation matrix–column pair (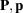) relating the basis of the subperiodic group 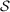 with the subgroup 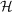. Note that different transformation matrices might specify the *same* (identical) subgroup, if these transformation matrices are related by an element of the affine normalizer 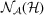 of the subgroup 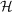. In other words, two subgroups of the same type 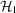 and 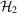 of 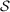 defined by the transformation matrix–column pairs (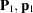) and (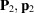) are identical if there is an element 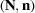 of the affine normalizer of the subgroup 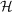 such as
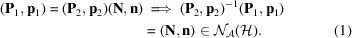
The Euclidean and affine normalizers of subperiodic groups are tabulated and available from VanLeeuwen *et al.* (2015 ▸).

As an example, let us consider the maximal subgroup *c*222 (No. 22) of index 2 of the layer group 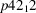 (No. 54). The transformation matrices describing this group–subgroup relation in Litvin’s book and the BCS are (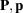)_Litvin_ = 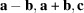; 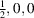 and (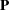, 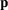)_BCS_ = 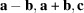; 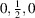, respectively. The affine normalizer 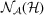 of the layer group *c*222 (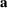, 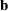, 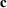) is the space group 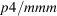 with basis vectors (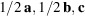). Applying equation (1), the translation 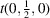 is obtained. Since 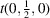 is an element of the affine normalizer of *c*222, the transformation matrices (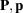)_Litvin_ = 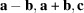; 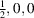 and (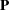, 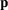)_BCS_ = 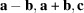; 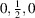 are equivalent and thus describe the *same* identical subgroup.

In general, the differences in (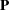, 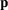) are due to the use of different conventions. For the maximal subgroups of rod groups belonging to trigonal or hexagonal groups with two descriptions in *IT*E, Litvin’s book prefers the use of the transformation 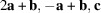; 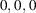 (found as the first option in Table 1.2.6.3 of *IT*E), while the BCS prefers the transformation 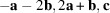; 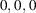 (second option in Table 1.2.6.3 of *IT*E). In the case of layer groups with two origins, these are described with respect to *origin choice 1* in Litvin’s book and *origin choice 2* in the BCS. Therefore, the information on maximal subgroups in these cases differs, since the information provided by these two sources corresponds to different settings.

### 5.2. List of errors found in Litvin’s book

As a result of the comparison of the two sources, a few errors were detected in the description of the maximal subgroups of rod and layer groups in Litvin’s book. Three types of errors were identified: (i) typographical errors (see Table 3), (ii) missing subgroups (see Table 4) and (iii) invalid transformation matrix–column pairs (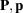) (see Tables 5 and 6).

#### 5.2.1. Typographical errors

Several typographical errors were found in the transformation matrices (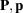) relating the subgroup *cm*2*m* (No. 35) of the layer group 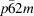 and the subgroups 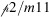 (No. 6), 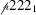 (No. 14) and 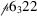 (No. 65) of the rod groups 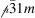 (No. 51), 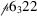 and 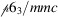 (No. 75), respectively. In these cases, four entry transformation matrices are provided (see Table 3 ▸) to relate the bases of these groups with their maximal subgroups. These are clear typographical errors in Litvin’s book.

Another typographical error can be found in the Hermann–Mauguin symbol of the only maximal subgroup of index 3 of the layer group 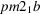 (No. 28). This subgroup corresponds to an isotypic subgroup of the group 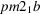; therefore, the symbol of the subgroup cannot be *pm*2*m* (No. 25) but should be 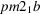.

#### 5.2.2. Missing subgroups

Among the maximal subgroups listed in Litvin’s book for the 80 layer, 75 rod and seven frieze groups, only a total of five maximal subgroups are missing for the rod groups 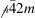 (No. 37) and 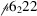 (No. 64) (*cf.* Table 4 ▸). There are two maximal subgroups 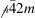 of index 2 for the rod group 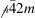:

[2] **c**′ = 2**c**.


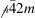
 (No. 37) 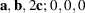.


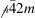
 (No. 37) 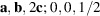.

In Litvin’s book, however, only the maximal subgroup with transformation matrix 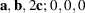 is listed. For the rod group 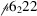, there are also two maximal subgroups 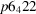 (No. 66) of index 2:

[2] **c**′ = 2**c**.


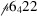
 (No. 66) 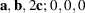.


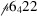
 (No. 66) 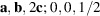.

In this case, the subgroup with transformation matrix 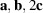; 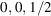 is not mentioned. The three conjugated subgroups 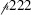 (No. 13) of the rod group 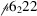 of index 3 are also missing from Litvin’s book.

#### 5.2.3. Invalid transformation matrix–column pairs (**P**, **p**)

Several cases can be found in Litvin’s book in which either the linear part 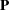 or the origin shift 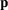 of the transformation matrix–column pair (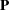, 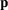) relating the basis of the group with the subgroup are not valid (see Tables 5 ▸ and 6 ▸). A non-zero origin shift 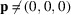 is defined in Litvin’s book for the transformation matrix relating the maximal subgroup *cmm*2 (No. 48) of the layer group 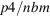 (No. 62) and the maximal subgroups 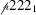 (No. 14), 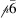 (No. 59), 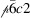 (No. 72) and 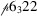 (No. 65) of the rod groups 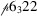 (No. 65), 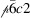 (No. 72), 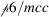 (No. 74) and 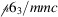 (No. 75), respectively. The non-zero origin shifts shown in Table 5 ▸ (column six) are not valid, since they do not properly describe their corresponding group–maximal-subgroup relation. In all these cases, it is necessary to have an origin shift 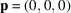. The transformation matrix relating the maximal subgroup 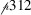 (No. 46) with the rod group 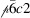, defined in Litvin’s book with a zero origin shift, is also not valid. The problem is again in the origin shift, which instead of zero is 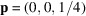. The origin shift of the transformation matrix describing the relation between the maximal subgroup *pb*2*b* (No. 30) of the layer group *pb*2*n* (No. 34) is not 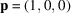, but 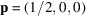.

There are only a few maximal subgroups of rod groups in Litvin’s book in which the linear part 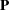 of the transformation matrix is not correctly defined (see Table 6 ▸). The transformation matrix 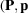)_Litvin_ = 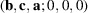, provided by Litvin’s book, describes the relationship between the maximal subgroups 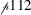 (No. 8) and 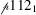 (No. 9) of index 3 of the rod groups 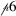 (No. 53) and 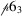 (No. 56), respectively. This transformation matrix results in different maximal subgroups: 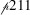 for 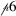, and 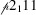 for 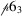 (No. 53). The valid transformation matrix for these cases requires a linear part 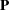 equal to the identity matrix, *i.e.*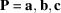. The linear part 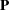 of the transformation matrix–column pairs of the three conjugated maximal subgroups 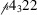 (No. 33) of index 3 for rod group 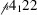 (No. 31), defined in Litvin’s book as 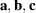, is also not valid. In this particular case, the correct 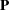 is 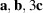. Similar problems (see Table 6 ▸) can also be found for the maximal subgroups 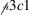 (No. 50) and 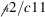 (No. 7) of index 3 and 2 of the rod group 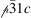 (No. 52).

## 6. Conclusions

The Bilbao Crystallographic Server offers the only complete and freely accessible database of maximal subgroups of subperiodic groups through the program *MAXSUB*. This program provides detailed information on both maximal non-isotypic and isotypic subgroups with indices up to 9, along with series of maximal isotypic subgroups.

A thorough comparison with the existing reference by Litvin (2013 ▸) has been conducted, revealing several discrepancies (which are analysed). These findings underscore the completeness of the BCS data, reinforcing their value as the most comprehensive resource for crystallographic research and subgroup analysis.

## Figures and Tables

**Table 1:** Maximal subgroups of indices up to 4 for the space group *P*4 (No. 75) Subgroups marked with *t* are *translationengleiche* and those marked with *k* are *klassengleiche*.

Group	Index	Type	( 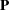 , 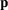 )
*P*112 (No. 3, *P*2)	2	*t*	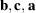
*P*4 (No. 75)	2	*k*	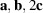
			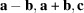
			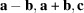 ; 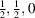
*P*4 (No. 75)	3	*k*	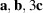
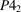 (No. 74)	2	*k*	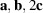
*I*4 (No. 79)	2	*k*	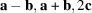
			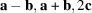 ; 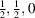

**Table 2:** Series of maximal isomorphic subgroups of the space group *P*4 (No. 75) For each of the series, the Hermann–Mauguin symbol of the subgroup, the index, the transformation matrix (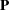, 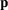) relating the group and the subgroup, and the restriction conditions on the parameters describing the series are provided.

	Subgroup	Index	( 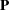 , 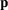 )	Conditions
Series #1	*P*4 (No. 49)	*p*	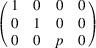	*p* prime
Series #2	*P*4 (No. 49)	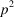	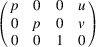	prime 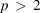
				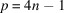
				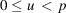
				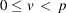
Series #3	*P*4 (No. 49)	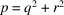	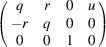	prime 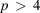
				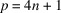
				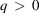 ; 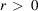
				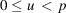

**Table 3:** Typographical errors found in Litvin’s book related to the transformation matrix–column pairs (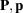) of the maximal subgroups of rod and layer groups The subgroups marked with *t* correspond to *translationengleiche* subgroups.

Group	Subgroup	Index	Type	( 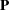 , 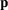 )
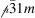 (No. 51)	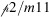 (No. 6)	3	*t*	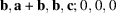
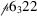 (No. 65)	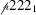 (No. 14)	3	*t*	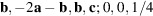
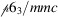 (No. 75)	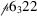 (No. 65)	2	*t*	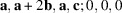
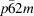 (No. 79)	*cm*2*m* (No. 35)	3	*t*	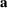 , 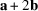 , 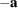 , 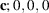

**Table 4:** List of the missing maximal subgroups of rod groups 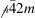 (No. 37) and 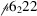 (No. 64) in Litvin’s book Subgroups marked with *t* are *translationengleiche* and those marked with *k* are *klassengleiche*.

Group	Subgroup	Index	Type	( 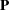 , 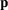 )
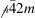 (No. 37)	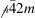 (No. 37)	2	*k*	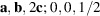
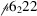 (No. 64)	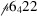 (No. 66)	2	*k*	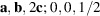
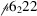 (No. 64)	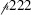 (No. 13)	3	*t*	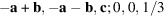
				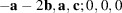
				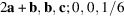

**Table 5:** Maximal subgroups of rod and layer groups and their corresponding transformation matrices (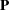, 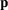) as listed in Litvin’s book, whose origin shift 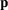 is invalid The valid origin shift 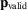 is given in the last column of the table. Subgroups marked with *t* are *translationengleiche* and those marked with *k* are *klassengleiche*.

Group	Subgroup	Index	Type	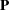	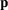	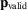
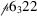 (No. 65)	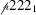 (No. 14)	3	*t*	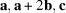	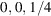	0, 0, 0
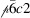 (No. 72)	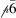 (No. 59)	2	*t*	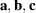	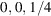	0, 0, 0
	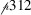 (No. 46)			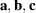	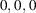	0, 0, 1/4
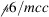 (No. 74)	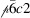 (No. 72)	2	*t*	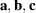	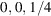	0, 0, 0
				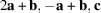	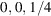	0, 0, 0
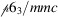 (No. 75)	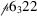 (No. 65)	2	*t*	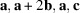	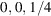	0, 0, 0
*pb*2*b* (No. 30)	*pb*2*n* (No. 34)	2	*k*	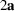 , 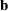 , 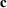	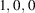	1/2, 0, 0
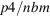 (No.62)	*cmme* (No. 48)	2	*t*	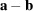 , 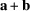 , 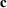	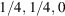	0, 0, 0

**Table 6:** List of the maximal subgroups of rod groups and their corresponding transformation matrices (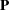, 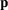) as listed in Litvin’s book, whose linear part 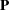 is invalid The correct linear part 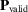 is given in the last column of the table. Subgroups marked with *t* are *translationengleiche* and those marked with *k* are *klassengleiche*.

Group	Subgroup	Index	Type	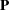	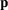	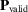
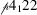 (No. 31)	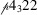 (No. 33)	3	*k*	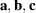	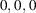	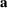 , 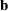 , 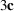
				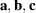	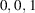	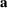 , 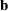 , 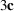
				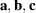	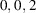	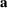 , 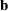 , 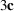
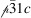 (No. 52)	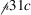 (No. 50, 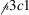 )	2	*t*	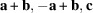	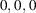	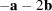 , 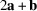 , 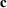
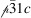 (No. 52)	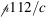 (No. 7, 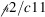 )	3	*t*	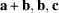	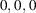	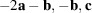
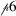 (No. 53)	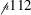 (No. 8)	3	*t*	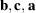	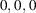	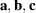
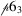 (No. 56)	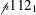 (No. 9)	3	*t*	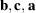	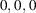	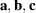

## Appendix A Types of subgroups of subperiodic groups

On the basis of Hermann’s theorem (Hermann, 1929 ▸) for space groups, the following types of subgroups of subperiodic groups can be distinguished:

(i) A subgroup  of a subperiodic group  is called a *translationengleiche* subgroup or a *t* subgroup of  if the set  of translations is retained, *i.e.*, but the order of the point group  is reduced.

(ii) A subgroup  of a subperiodic group  is called a *klassengleiche* subgroup or a *k* subgroup if the set  of all translations of  is reduced to  but the point group  is the same as that of .

(iii) A *klassengleiche* or *k* subgroup  is called an isotypic subgroup if it belongs to the same affine subperiodic group as .

(iv) A subgroup of a subperiodic group  is called *general* or *a general subgroup* if it is neither a *translationengleiche* nor a *klassengleiche* subgroup, *i.e.* and .
